# Associations between responsive parental behaviours in infancy and toddlerhood, and language outcomes at age 7 years in a population‐based sample

**DOI:** 10.1111/1460-6984.12846

**Published:** 2023-02-01

**Authors:** Penny Levickis, Patricia Eadie, Fiona Mensah, Cristina McKean, Edith L. Bavin, Sheena Reilly

**Affiliations:** ^1^ Melbourne Graduate School of Education The University of Melbourne Melbourne, VIC Australia; ^2^ Genetics Murdoch Children's Research Institute Melbourne, VIC Australia; ^3^ Intergenerational Health Murdoch Children's Research Institute Parkville VIC Australia; ^4^ Department of Paediatrics University of Melbourne Parkville VIC Australia; ^5^ School of Education, Communication & Language Sciences Newcastle University Newcastle upon Tyne UK; ^6^ School of Psychology and Public Health La Trobe University Melbourne VIC Australia; ^7^ Health Group Griffith University Gold Coast QLD Australia

**Keywords:** early childhood, language development, longitudinal studies, parent–child interaction

## Abstract

**Background:**

A wealth of evidence supports the important role high‐quality parent–child interactions play in children's early language acquisition. However, the impact on later language outcomes remains unclear.

**Aims:**

To examine the associations between responsive parental behaviours across the early years and child language outcomes at age 7 years with families from an Australian longitudinal cohort study (*N* = 1148, 50% female).

**Methods & Procedures:**

At child ages 12, 24 and 36 months, parents completed a self‐report measure of responsive parental behaviours. Child language was directly assessed at age 7 using the Clinical Evaluation of Language Fundamentals, 4th edition (CELF‐4), Australian Standardisation. Linear regression was used to examine associations between responsive parental behaviours from 12 to 36 months (consistently high, inconsistent and consistently low responsive parental behaviours at the three time points) and language scores at age 7 years. Adjusted models were run, including the following potential confounders: child sex; birth weight; birth order; maternal education; socio‐economic disadvantage; non‐English‐speaking background; family history of speech–language problems; mother's vocabulary score; maternal mental health score; and mother's age at birth of child. A final adjusted model was run, including the potential confounder variables as well as adjusting for children's earlier language skills.

**Outcomes & Results:**

Linear regression results showed children with parents who rated high on responsive parental behaviours at all three time points had higher mean language scores at age 7 than children whose parents reported low responsive parental behaviours across early childhood. This association attenuated after adjusting for earlier child language skills.

**Conclusions & Implications:**

Findings support the consistent use of responsive parental behaviours across the very early years of childhood to support long‐term language outcomes. Findings also suggest that models of surveillance and support which monitor and assist families at multiple time‐points over the early years are likely to be most effective for preventing ongoing language difficulties.

**WHAT THIS PAPER ADDS:**

**What is already known on this subject**

There is extensive evidence consistently demonstrating the important contribution of aspects of parent–child interaction, specifically responsive parental behaviours, to children's language development.

**What this paper adds to the existing knowledge**

Understanding the cumulative benefit of responsive parent–child interactions across the very early years may help to inform preventive interventions and service delivery models for supporting young children's language development. This study demonstrates in a large, population‐based cohort the contribution of consistency of responsive parental behaviours during infancy and toddlerhood to school‐age language outcomes, accounting for other child, family and environmental factors. Capturing regular parent behaviours via self‐report during the early years may be a more efficient and less costly method than parent–child interaction observations to monitor the home language‐learning environment during routine developmental checks.

**What are the potential or actual clinical implications of this work?**

Findings support the need for surveillance of children and families in the early years, ensuring that intervention occurs when families need it most, that is, support is responsive to changing needs and that nuanced advice and support strategies are provided to activate positive developmental cascades. Capturing both parent behaviours and child language may assist clinicians to identify those families who may benefit from parent–child interaction intervention.

## INTRODUCTION

Language acquisition is a critical part of a child's overall development, central in learning, socializing and forming relationships. Early language skills are a strong predictor of school readiness and later academic and social skills (e.g., Bleses et al., 2016; Eadie et al., 2021). Therefore, any delays in a child's early language development are a cause of concern for parents, caregivers and health professionals. While language delays in early childhood are common, high levels of resolution and fluctuation make it difficult to identify children who will go on to have persistent language difficulties (Reilly et al., 2010; Zambrana et al., 2014). Children with persistent language difficulties are at risk of poorer mental health, lower self‐esteem, poorer academic performance and employment opportunities, and lower quality of life than their typically developing peers (Eadie et al., 2018; Johnson et al., 2010; Schoon et al., 2010). The prevalence of children with language difficulties when they begin school is even greater for children from socio‐economically disadvantaged backgrounds (Law et al., 2011). One factor known to influence language development is a child's language‐learning environment. Specifically, the amount and quality of interaction between parent and child has consistently been shown to be related to children's language skills (Hirsh‐Pasek et al., 2015; Madigan et al., 2019; Rowe, 2012) and as such has been the focus of many preventative intervention programmes around the world, particularly targeting socially disadvantaged families (Jeong et al., 2021; Levickis et al., 2020). While there is evidence supporting the short‐term benefits of responsive and contingent parent–child interactions to child language outcomes (Heidlage et al., 2020; McGillion et al., 2017), the extent to which these interactions are associated with longer term language outcomes remains unclear, as does the degree to which the *consistency* of responsive and contingent interactions over the pre‐school period matters. Understanding the contributions of parent–child interaction across the first few years of life to later school‐age outcomes could inform early interventions for promoting language acquisition in the preschool period.

### Features of parent–child interaction associated with child language development

Children require an optimal language‐rich environment to develop the necessary language skills to realize their full potential. Established theoretical perspectives, social interactionist theory (Bruner, 1981) and Vygotsky's (1978) zone of proximal development (ZPD), have contributed to understanding how child language develops within a social context that responds to children's current and emerging abilities. The concept of ZPD relates to adult linguistic input that scaffolds slightly above the child's level of development but is not beyond what a child can understand or how they use language. Both theories highlight that the social interactions that children experience with those around them are crucial to their language development; that the nature of these interactions is reciprocal, with children playing an active role; and that the most supportive form of adult input is that which is responsive and focused on the child's interests and needs (Rowe & Snow, 2020).

There is extensive evidence which consistently identifies the components of quality parent–child interaction shown to be associated with better language development (Ginsborg, 2006; Levickis et al., 2014; Madigan et al., 2019). Rowe and Snow (2020) recently conceptualized the features of caregiver input that facilitate language development in terms of three dimensions of input quality: interactive, linguistic and conceptual. Interactive features include responsiveness and shared attention; linguistic features include levels of linguistic complexity and redundancy that are adapted to the child's developmental stage; and conceptual features include topics of conversation that provide appropriate challenges for the child's level of development. The types of responsive parent behaviours used in infancy and toddlerhood shown to predict child language outcomes include those that support the child to make connections between their own linguistic output or focus of attention and their parent's linguistic response (Yoder et al., 1998), as well as using rich verbal input in response to the child's communicative act (Tamis‐LeMonda et al., 2001).

Quantity of parent linguistic input to their young children has been extensively explored, highlighting a relationship between amount of parent linguist input and children's language outcomes (Hart & Risley, 1995; Hoff & Naigles, 2002; Huttenlocher et al., 1991). The literature also draws attention to the association with social disadvantage of both frequency of child‐directed speech and quality of input, with lower frequency and lower quality tending to occur in families living with social disadvantage when compared with those children from more advantaged backgrounds (e.g., Hart & Risley, 1995; Sultana et al., 2020). It is important to note that while socio‐economic background predicts group‐level differences in parents’ language input and children's language skills, there is a large degree of variability between families within socio‐economic groups regarding both the quantity and quality of parent–child interactions (Schwab & Lew‐Williams, 2016). Whilst a social gradient exists, it is clearly not the case that every child experiencing social disadvantage encounters a less‐than‐optimal language‐learning environment.

A wealth of evidence supports the important role high‐quality parent–child interactions play in children's early language acquisition (for a recent meta‐analysis, see Madigan et al., 2019). In a longitudinal study of 50 parent–child dyads, Rowe (2012) found quantity of parent linguistic input was most important during the second year of life, while qualitative aspects of input, including different types of talk (such as rare word types and narrative utterances), were important during the third and fourth years of life. A study by Newman et al. (2016) examining the role of infant processing skills and child‐directed speech at age 7 months on language outcomes at age 2 years showed type–token ratio predicted children's vocabulary outcomes, that is, parents producing fewer different words but repeating them frequently had children with larger vocabularies later. Rowe and Snow (2020) argue that while parents’ frequent repetition of words in infancy predicts child language outcomes, this relationship becomes inverse as children get older, that is, as children get older if parents are continuing to use more repetition to support children's understanding this may be indicative of language difficulties. Aspects of parents’ communicative interactions with their child need to change as children's developmental abilities increase, although facets of shared attention and back‐and‐forth non‐verbal and verbal exchanges are ongoing in language‐promoting interactions (Rowe & Snow, 2020). The developmental progression and need for a parent to adjust their interaction style to meet their child's stage of development may be a contributing factor to the diminishing long‐term effects of parent–child interaction language interventions (McGillion et al., 2017; Suskind et al., 2016). As highlighted by Rowe and Snow (2020), the most important parent linguistic input during parent–child interactions across early childhood is characterized by responsiveness and shared attention, input that is linguistically adapted to the child's developmental level and conceptually challenging. Henceforth, this type of parent linguistic input is referred to as responsive parental behaviours.

### Consistency of quality parent–child interactions across early years of development

While evidence suggests that quality parent–child interactions in early childhood are associated with better short‐ and potentially long‐term language outcomes, more evidence is required to understand whether any exposure to quality parent–child interactions is beneficial or whether consistency (i.e., quality parent–child interactions throughout stages of early language development) is key and at which stage is most critical. If consistency is important, there is an argument that interventions should provide parents with specific developmental stage‐related strategies that scaffold language skills at different times throughout early childhood. This maintenance is particularly pertinent given findings of ‘wash‐out’ from initial intervention gains. Intervention at one time point may make a difference for a short period but may not result in ongoing language‐promoting interactions that result in sustained improvement in language outcomes over time (McGillion et al., 2017).

Responsive parental behaviours are shown to be as important to supporting language acquisition during infancy as during the toddler period (Rowe & Snow, 2020). Consistent responsiveness across the early developmental periods may be required to assist children to maintain a positive trajectory of language acquisition due to the rapidly changing nature of these skills in the first few years of life (Landry et al., 2003). Drawing on a developmental cascade model of development, language outcomes that are observed in later childhood may be a reflection of the cumulative impact of reciprocal interactions between child, parent and environmental factors during earlier time points (Asmussen et al., 2018). It is hypothesized that when parents provide children with language rich home learning environments, these early experiences will promote children's development of foundational language skills that activate developmental cascades (Tamis‐LeMonda et al., 2019). This would suggest that responsive parental behaviours in the very early years may benefit children's language development to the extent that a child develops sufficiently robust early language skills and can ‘drive’ language‐learning interactions, furthering their lexical and grammatical growth. However, this is not to say that if children experience suboptimal language environments in the early years, they cannot ‘catch up’, but the risk of poorer outcomes increases for children who are exposed to less stimulating home environments for ongoing periods of time (Asmussen et al., 2018). Further supporting the important contribution of early parent–child interaction as a risk factor for later language difficulties are findings from a recent study examining the association between cumulative and clustering risk factors in the early years and school‐age language outcomes (Eadie et al., 2022). Eadie et al. (2022) describe a number of subgroups characterized by differing clustering of risk factors. Those subgroups which were vulnerable to poorer language outcomes consistently included lower parent–child interaction scores compared with a developmentally enabled subgroup with typical language development.

Consistency of children's home learning environment, including parent–child interactions, and early learning experiences that are stable over time, and responsive to a child's developmental level could therefore contribute to longer term positive outcomes (Tamis‐Lemonda et al., 2019). Landry et al. (2003) examined the relationship of consistency of maternal responsiveness across early childhood (birth to 4 years of age) with children's language and social outcomes by age 8 years in a sample of preterm and term children. Findings showed children who experienced consistently high levels of maternal responsivity across the early years, particularly preterm children, scored higher on language ability compared with those children who experienced high responsivity in either the earlier years or later years, but not consistently. While this study provides some evidence suggesting consistency of responsiveness during early childhood is important for language skills, generalizability of findings is limited as the sample of ‘term’ children included is homogenous and a limited number of covariates were included. It is important to determine whether relationships between consistency of responsiveness and child language outcomes are evident after accounting for a range of child, family, and environmental factors, and examining this in a representative, population‐based sample. If parent–child interactions consistently characterized by responsive parental input during the early years are shown to be associated with better school‐aged language skills, this could inform policy and practice, suggesting the need for a series of language promoting early interventions, rather than a single intervention at a particular age or stage of development.

### Parent–child interaction and school‐aged language outcomes

The work by Landry et al. (2003) supports the idea that early quality parent–child interactions could catalyse positive developmental cascades supporting longer term robust language development beyond the early years. However, relatively few studies have examined this issue. In a longitudinal study using the sample originally included in the Hart and Risley study, Walker et al. (1994) examined whether the associations found by 36 months of age between family socio‐economic status (SES), parenting and early language development would persist and be identifiable in later school age language outcomes. In the original study (Hart & Risley, 1995), children with smaller vocabularies at age 36 months were from more disadvantaged backgrounds and their parents were observed to use less varied vocabularies during parent–child interactions, to interact less often with their children and ask fewer questions. Results indicated that the rate of vocabulary growth from 7 to 36 months of age predicted receptive vocabulary and receptive and expressive language at age 9–10 years. Walker et al (1994) found that for this sample of children, SES‐related differences (measured by occupational status, family income and mother's educational level) at 36 months in child language prior to school predicted verbal ability, spoken and receptive language and academic achievement assessed using standardized measures at 9–10 years of age. Authors concluded that the findings support the notion that children of socially disadvantaged backgrounds experience less rich home language environments associated with poorer language outcomes, and therefore cumulative language spoken in the home is an important predictor of later language and academic outcomes.

In one of the few studies exploring the long‐term association between parent–child interactions in early childhood and later language skills, Gilkerson et al. (2018) collected daylong audio recording of 2–36‐month‐old children and examined the association between total number of daily adult words and adult–child conversational turns (analysed using Language Environment Analysis software) and language skills 10 years later. The analysis included examining language exposure (using an automated method to estimate Conversational Turn Counts and Adult Word Counts) according to three age groups: 2–17, 18–24 and 25–36 months. A significant relationship was found for the 18–24 months age group, with the average Conversational Turn Counts (vocal initiations with responses that occur within 5 s) and Adult Word Counts (includes both overheard and child‐directed speech) predicting child language outcomes. However, after controlling for maternal education (used as a marker for SES), Conversational Turn Counts continued to predict language outcomes, but the association between Adult Word Counts and language outcomes weakened significantly. While this study demonstrates that conversational turns, a feature of parent–child interactions, used during toddlerhood (18–24 months) may contribute to better long‐term language outcomes, the question still remains as to whether consistent use of responsive parental behaviours across early childhood matters. This could be addressed by examining the consistency of responsiveness in a large population‐based cohort, including multiple data collection points of parent–child interaction across the early years, direct assessment of child language outcomes, as well as the capability to account for other child, family and environment factors that may also contribute to child language outcomes.

### The present study

Further research is required examining the long‐term relationship between early parent–child interactions characterized by responsive parental behaviours and later language outcomes. In addition, understanding the cumulative benefit of responsive parental behaviours across the very early years may help to inform preventive interventions and service delivery models for supporting young children's language development.

Using data from the Early Language in Victoria Study (ELVS), we aimed to examine associations between consistently high, inconsistent and consistently low responsive parental behaviours across 12, 24 and 36 months, and language outcomes at age 7 years.

## METHODS

### Design and participants

This study uses data from a prospective, longitudinal cohort study of language development from infancy to adolescence (Reilly et al., 2018). Between September 2003 and April 2004, the ELVS team recruited 1910 infants aged between 7.5 and 10 months of age from six local government areas (LGAs) in Melbourne, Australia. LGAs were selected to represent the geographic and socio‐economic spectrum using the census‐based Socio‐Economic Index of Areas (SEIFA) (Australian Bureau of Statistics, 2001). Exclusion criteria included children with a serious disability or developmental delay at birth (e.g., Down syndrome, hearing impairment), and parents with insufficient English to complete written questionnaires without an interpreter. The total uptake rate for the study was 82% (1910/2335). Further detail of the sampling and recruitment process is extensively reported elsewhere (Reilly et al., 2018, 2010). The data utilized in the current study were collected via parent surveys at child ages 12, 24 and 36 months. Outcome data were collected at age 7 years, either in the home or at school, in which trained researchers conducted face‐to‐face language assessments with the children.

All participants provided written informed consent. Ethics approval was received from The Royal Children's Hospital Human Research Ethics Committee (27078/33195) and the La Trobe University Human Ethics Committee (03‐32).

### Measures

#### Predictor: Parent–child interaction measure

The Brigance Parent–Child Interactions Scale (BPCIS) is an 18‐item parent‐reported measure of parenting behaviours and parents’ perceptions about their child, drawn from relevant literature demonstrating parenting interaction styles and behaviours associated with language development (Glascoe, 2002; Glascoe & Leew, 2010). A total of 10 items from the BPCIS were included in the ELVS parent questionnaire at ages 12, 24 and 36 months to capture parent behaviours expected to be associated with language development, such as ‘I help my child learn by talking and showing him or her new things’ (Glascoe & Leew, 2010). For the current study, the eight BPCIS items that reflect positive parent behaviours were included in the analysis as these reflect aspects of possessive, i.e., Rowe and Snow's (2020) interactional feature of caregiver input quality, such as responsiveness and responding contingently to a child's needs and interests (e.g., ‘When my child seems interested in things, I talk to him/her about his or her interests’) (Table 1). Each item is scored a 1, 2 or 3 for the options not very often, sometimes and often, respectively. Therefore, participants may receive a minimum score of 8 and a maximum score of 24. The two negative items ‘My child is not very much fun to be with’ and ‘When my child looks at or touches something, the first thing I say is “no”’ were excluded as the focus is positive, responsive parental behaviours shown to be associated with better child language outcomes. Cronbach's alpha was also calculated to confirm the reliability of the eight selected items, with the value increasing from 0.69 for the 10 items to 0.72 for the eight items.

**TABLE 1:** Items from the parent‐reported Brigance Parent–Child Interaction Scale (BPCIS) included in parent questionnaires at child ages 12, 24 and 36 months

	**Response options, frequency at each age (%)**								
	**Not very often**			**Sometimes**			**Often**		
BPCIS item	**12 months**	**24 months**	**36 months**	**12 months**	**24 months**	**36 months**	**12 months**	**24 months**	**36 months**
I play with my child and show him/her things	0.89	0.53	0.54	20.54	15.79	26.43	78.57	83.67	73.03
I help my child learn by talking and showing him/her new things	0.54	0.44	0.45	21.16	13.31	19.89	78.30	86.25	79.65
I look at or read children's books to my child	11.44	1.60	2.73	39.59	17.94	16.38	48.97	80.46	80.89
When my child seems interested in things, I talk to him/her about his or her interests	5.37	1.15	0.64	38.01	21.58	19.13	56.62	77.26	80.24
I talk to my child in a special way	12.73	17.66	26.22	35.47	36.76	40.75	51.81	45.59	33.03
Most of the time I enjoy my child	0.99	1.60	1.00	1.97	2.75	6.63	97.04	95.65	95.37
I make up games or songs for my child	7.60	8.16	10.68	39.95	43.30	46.88	52.46	48.54	42.44
When my child looks at/touches a toy, I talk to him/her about it	4.92	2.49	5.98	47.41	43.96	53.26	47.67	53.55	40.76

*Note*: Response codes: not very often = 1, sometimes = 2 and often = 3.

#### Outcome: Language measure

Trained research assistants conducted assessments with children using the Clinical Evaluation of Language Fundamentals, 4th edition (CELF‐4), Australian Standardisation (Semel et al., 2006) at age 7 years. The CELF‐4 produces a core language standard score, with a mean of 100 and standard deviation (SD) of 15.

#### Potential confounding variables

Potential confounders of the association between parent–child interaction and child language were specified a priori on the basis of demonstration of association with language outcomes in previous literature and being shown to predict child language in the ELVS sample previously (Reilly et al., 2010). These include: child sex; birth weight; birth order; maternal education; the Socio‐Economic Indexes for Areas (SEIFA) disadvantage index score (Australian mean = 1000; SD = 100) as a proxy for SES; non‐English‐speaking background (defined as ‘main language spoken to the child in the home’); family history of speech–language problems; mother's vocabulary score; maternal mental health score and mother's age at birth of child. SEIFA scores are derived from the Australian Census, with a higher score indicating a less disadvantaged neighbourhood relative to other areas (Australian Bureau of Statistics, 2001). Maternal mental health was measured using the self‐report Nonspecific Psychological Distress Scale (K‐6) (Kessler & Mroczek, 1994), dichotomized as ‘likely mental distress’ (score of ≥ 4 of a possible 24) versus ‘no mental distress’ (score of <4). This is a low threshold cut point to identify those mothers with any self‐reported indication of mental distress from those with no indication of mental distress.

#### Early language measure

Early communication skills are a strong predictor of later language ability, so a measure of early expressive vocabulary was additionally considered to explore whether children's language skills may influence parent–child interaction and may then predict later language outcomes. At 24 months of age, parents completed the MacArthur–Bates Communicative Development Inventory, words and sentences. Expressive vocabulary production scores (raw scores) were included in the analyses.

### Analysis

First, we created a correlation matrix to examine the correlation between the eight BPCIS items at each age (12, 24 and 36 months) and language scores at age 7 years (Table 2). Language outcomes at age 7 years were positively correlated with each of the BPCIS items at each time point. Next, we dichotomized the BPCIS total raw score at each wave to create a high versus low responsive parental behaviour variable at each time point. Descriptive statistics of the total BPCIS score were examined to check the distribution, mean, median and SD at each time point. The median was determined to be an appropriate cut point as there are no recognized cut points for the BPCIS (median and range at 12, 24 and 36 months, respectively: 21, 17 and 16). From this, a categorical variable using the dichotomized BPCIS from each time point was generated and labelled ‘responsive parental behaviours consistency variable’. The groups in this variable were categorized as: consistently high responsive parental behaviours score (at or above the median BPCIS total score at three time points); inconsistent responsive parental behaviours score (at or above the median BPCIS total score at one or two time points); and consistently low responsive parental behaviours score (below the median BPCIS total score at three times points).

**TABLE 2:** Correlation matrix—BPCIS at each age (12, 24 and 36 months) and CELF Core Language standard score at age 7 years

	**BP1** **12, 24 and 36 months**	**BP2** **12, 24 and 36 months**	**BP3** **12, 24 and 36 months**	**BP4** **12, 24 and 36 months**	**BP5** **12, 24 and 36 months**	**BP6** **12, 24 and 36 months**	**BP7** **12, 24 and 36 months**	**BP8** **12, 24 and 36 months**	**CORE 7**
BP1	1.00								
BP2									
12 months	0.58^c^	1.00							
24 months	0.68^c^	1.00							
36 months	0.62^c^	1.00							
BP3									
12 months	0.38^c^	0.36^c^	1.00						
24 months	0.39^c^	0.41^c^	1.00						
36 months	0.33^c^	0.26^c^	1.00						
BP4									
12 months	0.34^c^	0.43^c^	0.41^c^	1.00					
24 months	0.40^c^	0.42^c^	0.35^c^	1.00					
36 months	0.28^c^	0.33^c^	0.25^c^	1.00					
BP5									
12 months	0.11^c^	0.15^c^	0.14^c^	0.22^c^	1.00				
24 months	0.11^c^	0.13^c^	0.13^c^	0.17^c^	1.00				
36 months	0.11^c^	0.13^c^	0.13^c^	0.17^c^	1.00				
BP6									
12 months	0.02	0.02	0.06	0.02	0.04	1.00			
24 months	0.06^a^	0.06^b^	0.11^c^	0.06^a^	0.04	1.00			
36 months	0.06^a^	0.06^a^	0.11^c^	0.06^a^	0.04	1.00			
BP7									
12 months	0.25^c^	0.30^c^	0.21^c^	0.26^c^	0.1460^c^	0.09^b^	1.00		
24 months	0.31^c^	0.29^c^	0.25^c^	0.33^c^	0.16^c^	0.09^b^	1.00		
36 months	0.31^c^	0.29^c^	0.25^c^	0.33^c^	0.16^c^	0.09^b^	1.00		
BP8									
12 months	0.38^c^	0.44^c^	0.33^c^	0.52^c^	0.23^c^	0.02	0.38^c^	1.00	
24 months	0.32^c^	0.33^c^	0.30^c^	0.47^c^	0.20^c^	0.06^b^	0.44^c^	1.00	
36 months	0.32^c^	0.33^c^	0.30^c^	0.47^c^	0.20^c^	0.06^b^	0.44^c^	1.00	
CORE 7	0.10^c^	0.10^c^	0.20^c^	0.19^c^	0.07^a^	0.07^a^	0.06	0.13^c^	1.00
	0.14^c^	0.14^c^	0.21^c^	0.18^c^	0.06	0.14^c^	0.12^c^	0.17^c^	1.00
	0.14^c^	0.14^c^	0.21^c^	0.18^c^	0.06	0.14^c^	0.12^c^	0.17^c^	1.00

*Notes*: BP, Brigance Parent–Child Interactions Scale (BPCIS) (items 1–8); CORE 7, CELF Core Language standard scores at age 7.

^a^ *p* < 0.05;

^b^ *p* < 0.01;

^c^ *p* < 0.001.

To address our primary aim to examine the association between parent–child interaction (specifically, responsive parental behaviours) across 12–36 months of age and language outcomes at ages 7 years, we ran unadjusted linear regression models, including the responsive parental behaviours consistency variable and child language outcome at age 7 years. Next, adjusted models were run, which included the following potential confounders: child sex; birth weight; birth order; maternal education; SEIFA Index for Relative Socio‐Economic Disadvantage score; non‐English‐speaking background; family history of speech–language problems; mother's vocabulary score; maternal mental health score; and mother's age at birth of child. A final adjusted model was run, including the potential confounder variables as well as adjusting for children's earlier language (parent‐reported expressive vocabulary at age 24 months). We adjusted for earlier language skills to address the issue that children's language skills may be driving the parent–child interaction which may then predict later language outcomes. All analysis was conducted using Stata version 16.0 (StataCorp, 2019).

## RESULTS

Of the original ELVS sample, 1148 of 1910 (60.1%) contributed parent–child interaction data and child language outcome data used in the current analyses. Table 3 shows the sample characteristics of those participants who provided data included in the current analyses compared with those who did not. Compared with those not included, a higher proportion of children included in the analyses had mothers who had completed 13 years of schooling or a degree/postgraduate degree (80% versus 71%) and had a slightly higher mean SEIFA disadvantage score (1042 versus 1027). The sample of children included in the current analysis had, on average, 20 words more at age 2 than those who did not provide data at age 7 years. A high proportion of children included in the current analysis spoke English only at ages 24 and 36 months (96% and 97%, respectively) and half the sample was female. Almost a third of mothers (32%) reported symptoms indicative of any mental distress at child age of 12 months. The mean and SD of language score for children in the current analysis were 98.0 and 13.3, respectively.

**TABLE 3:** Comparing participant characteristics of the whole ELVS cohort with those who provided data at age 7 and were included in the current study

**Item**	**Participating at age 7** *	**Not participating at age 7**
*n*, (%)	1148 (60.10)	762 (39.90)
*Child sex*		
Female	587 (50.13)	358 (46.98)
*Birth order*		
1st	591 (51.53)	362 (47.95)
2nd	385 (33.57)	287 (38.01)
3rd or more	171 (14.91)	106 (14.04)
*Maternal education*		
Year 10 or less	79 (6.90)	100 (13.16)
Year 11	150 (13.10)	118 (15.53)
Year 12	448 (39.13)	317 (41.71)
Degree or postgraduate qualification	468 (40.87)	225 (29.61)
*Non‐English‐speaking background (at 2 years)*		
English only	1085 (96.19)	539 (89.39)
Bilingual English and another language	8 (0.71)	16 (2.65)
Only language other than English	35 (3.10)	48 (7.96)
*Non‐English‐speaking background (at 3 years)*		
English only	1057 (97.15)	493 (92.67)
Bilingual English and another language	8 (0.74)	9 (1.69)
Only language other than English	23 (2.11)	30 (5.64)
*Family history of speech–language problems*		
No history	872 (75.96)	563 (73.88)
History	276 (24.04)	199 (26.12)
*Maternal mental health problems*		
Indicated	358 (32.05)	197 (31.07)
Not indicated	759 (67.95)	437 (68.93)
*Mean (SD)*		
Birth weight (g)	3463.35 (515.34)	3382.55 (560.70)
Index of disadvantage—SEIFA	1041.79 (54.95)	1027.39 (67.66)
Maternal age at birth of child (years)	32.18 (4.34)	31.34 (4.81)
Maternal vocabulary score	28.19 (4.61)	26.31 (5.64)
BPCIS total score—12 months	20.67 (2.57)	20.23 (2.75)
BPCIS total score—24 months	21.37 (2.31)	20.95 (2.40)
BPCIS total score—36 months	20.78 (2.31)	20.63. (2.33)
Expressive vocabulary at 24 months	268.47 (159.66)	246.47 (166.46)

*Note*: ^*^Sample size ranges from 1122 to 1148.

The correlation matrix of the eight BPCIS items at each time point (12, 24 and 36 months) and child language scores at age 7 showed that all BPCIS items were associated with language outcomes (Table 2). However, the strength of these associations was weak with correlations ranging from 0.1 to 0.2. The average BPCIS total score at each time point were as follows: 20.7 (SD = 2.6, range = 11–24) at 12 months; 21.4 (SD = 2.3, range = 10–24) at 24 months and 20.8 (SD = 2.3, range = 11–24) at 36 months. The proportions of participants in each category of the responsive parental behaviours consistency variable created to include in the regression analyses were: 22.7% ‘consistently low’; 42.5% ‘inconsistent’; and 34.9% ‘consistently high’.

The results from unadjusted and adjusted linear regression models including the responsive parental behaviours consistency variable and child language outcomes at age 7 years are presented in Table 4. In unadjusted models, children with parents reporting consistently high responsive parental behaviours and inconsistent responsive parental behaviours across ages 12, 24 and 36 months had mean language scores higher than the consistently low group at age 7 years. Children whose parents reported consistently high responsive parental behaviours at all three time points had a language score 0.5 SD higher than those children whose parents reported consistently low responsive parental behaviours. While regression coefficients attenuated after adjusting for potential confounder variables (child sex, birth weight, birth order, maternal education, SEIFA, non‐English‐speaking background, family history of speech–language problems, mother's vocabulary score, maternal mental health score and mother's age at birth of child), the consistently high responsive parental behaviours group was associated with an average language score around 0.5 SD higher compared with the consistently low responsive parental behaviours group (*β* = 7.3 [5.3, 9.4]). In the final fully adjusted model, which included early language skills (expressive vocabulary at 24 months) in addition to potential confounder variables, while the adjustment attenuated the estimate to some degree, there was still evidence that children in the consistently high responsive parental behaviours group had an average language score at age 7 slightly higher than children in the consistently low group (*β* = 2.5 [0.5, 4.5]). This suggests that early language skills may partially mediate the association between responsive parental behaviours and later language outcomes. The inconsistent responsive parental behaviours group had mean language scores lower than the consistently high responsive parental behaviours group, but higher than the consistently low group, although this difference was no longer evident by the third regression model, adjusting for confounders and earlier language skills. The proportion of variance explained at age 7 by the responsive parental behaviours consistency variable alone was 4.7%. This increased to 13.9% for the overall model after adjusting for potential confounder variables, and 23.4% in the fully adjusted model including earlier language skills.

**TABLE 4:** Unadjusted and adjusted associations between responsive parental behaviours consistency variable and CELF Core Score at age 7

	**Mean (SD)**	**Model 1** ***β* [95% CI], *p***	***R* ^2^ (%)**	**Model 2** ***β* [95% CI], *p***	***R* ^2^ (%)**	**Model 3** ***β* [95% CI], *p***	***R* ^2^ (%)**
CELF Core Score at age 7 years			4.36		12.99		22.33
*RPB consistency*							
Consistently high	94.15 (12.99)	Ref.		Ref.		Ref.	
Inconsistent	97.37 (13.61)	3.22 [1.25, 5.19], 0.001		3.20 [1.20, 5.20], 0.002		1.37 [−0.55, 3.30], 0.16	
Consistently low	101.47 (12.17)	7.32 [5.27, 9.37], < 0.001		5.22 [3.15, 7.28], < 0.001		2.47 [0.46, 4.47], 0.02	

*Note*: Model 1: unadjusted model; model 2: adjusted for potential cofounder variables including: child sex; birth weight; birth order; maternal education; SEIFA; non‐English‐speaking background; family history of speech–language problems; mother's vocabulary score; maternal mental health score and mother's age at birth of child; model 3: adjusted for covariates and earlier child language skills.

RPB, responsive parental behaviour variable.

## DISCUSSION

This large community‐based study showed that parent–child interactions, characterized by consistently high responsive parental behaviours across infancy and toddlerhood, are related to better child language outcomes in the early school years. Responsive parental behaviours measured at 12, 24 and 36 months were associated with child language outcomes at age 7 years. Specifically, children whose parents reported consistently high responsive parental behaviours at the three time points had later language scores that were higher than children whose parents reported inconsistent or consistently low responsive parental behaviours. After adjusting for both potential confounders and earlier language skills, these effects were largely explained by earlier language scores, although the consistently high responsive parental behaviours group continued to have higher average language scores than the consistently low group. The responsive parental behaviours variable continued to explain unique variance in children's school‐aged language outcomes after adjusting for potential risk factors and earlier language skills.

While the amount of variance explained by the responsive parental behaviours variable was relatively small, the variance explained by any one variable is often small and responsive parental behaviours are potentially one of the few mutable risk factors that can be addressed through intervention (McKean et al., 2015; Taylor et al., 2013). The small amount of variance explained may in part be due to a possible ceiling effect, with more parents self‐reporting higher positive responsive behaviours. It is also important to note that the current sample includes parents and children from higher SES backgrounds and mothers with higher education levels, which are factors associated with better language outcomes (Eadie et al., 2022), so associations for the responsive parental behaviours variable may in fact be understated. Thus, despite these limitations, findings do support the importance of consistency of responsive parental behaviours throughout early childhood (infancy and toddlerhood) and strongly support the need for early and sustained responsive behaviours and ways to support parents to provide these. While children's earlier language skills are an important contributing factor to children's later language outcomes, this study contributes to evidence suggesting that parent–child interaction needs to be included as part of early language intervention and promotion programmes (Eadie et al., 2022).

The current study confirms findings by Landry et al. (2003) who found that consistent responsiveness during early childhood (birth to 4.5 years) contributed to child language outcomes at 8 years. We extend these findings by demonstrating the relationship between consistently high responsive parental behaviours at different ages (12, 24 and 36 months) and language outcomes in early primary school in a large‐population‐based cohort, while uniquely contributing to the literature by accounting for a range of child, family and environmental factors.

While direct observations of parent–child interaction are commonly accepted as the gold standard (Bennetts et al., 2016), it is promising that the BPCIS parent‐report measure of responsive parental behaviours used in the current study could be used to determine consistency of responsive parental behaviours from 12 to 36 months, and this measure was associated with child language outcomes. Findings demonstrate potential for a parent‐report measure, such as BPCIS, to be used in large‐scale studies or clinically to capture evidence of responsive parental behaviours associated with language outcomes when it is not feasible to conduct direct observations. Items in the BPCIS speak to responsivity (e.g., talking to and showing a child new things) and shared attention (e.g., talking to child about what the child is interested in), which are well supported in the literature as predictors of language outcomes (Hart & Risley, 1995; Yoder et al., 1998). In addition, frequency of shared book reading is one of the BPCIS items (‘I look at or read children's books to my child’). Shared book reading provides an ideal context for contingent and responsive interactions to occur often including features such as talking, sharing, responsivity and reciprocity. Shared‐book‐reading may be one of the key items to include when measuring the home language‐learning environment, as well as an important strategy to target in parenting interventions. (Gilkerson et al., 2017; Niklas et al., 2016). However, focussing heavily on book‐reading could bias judgements where book reading is not part of a family's culture or experiences. Therefore, an easy to administer measure of responsive parental behaviours, such as the BPCIS, which includes a range of items may provide the opportunity to capture responsivity in other contexts.

In their systematic review and meta‐analysis, Jeong et al. (2021) showed that parenting interventions that target responsive parent–child interactions demonstrate a greater effect on child cognitive outcomes, parenting practices and parent–child interactions when compared with interventions that do not include a focus on responsive parenting. Findings from the current study further support and strengthen the argument that language‐promotion interventions should include a focus on enhancing responsive parental behaviours. As outlined by Rowe and Snow (2020), children's language skills develop rapidly during early childhood, and parents are required to adapt their input to support their child's changing abilities and provide learning experiences that are developmentally appropriate, challenging and help to scaffold children's language (Landry et al., 2003). This modification to the child's developmental level may be instinctive to some parents or a reflection of the childhood environment that parents experience or both. While some parents and their children may benefit from intervention at a particular stage of the child's language development, and can then go on to support their child successfully as they develop, others may require additional support to modify the nature of their input to ensure it is optimal for their child's language learning. A developmental cascade model suggest that skills beget skills, so as children acquire early language skills, this builds the foundations for further learning, literacy and later academic skills. This model can be used to explain, in part, why some children appear to acquire language with ease, as they can continue building on early established skills through language‐rich interactions, but also means that children who have not successfully acquired early language skills may not be able to catch up to their peers without additional support to activate developmental cascades (Tamis‐LeMonda et al., 2019). Furthermore, there are circumstances where the potential benefits of developmental cascades may be limited. Where children have biological risks which make language learning more difficult (such as those with a developmental language disorder) it is unlikely that a single parenting intervention focused on responsive behaviours that is not sustained will have sufficient cascading benefits to alleviate their difficulties. They will likely continue to find language learning a challenge and will need enhanced input to develop at a similar rate to their peers (Bishop et al., 2017). Furthermore, social disadvantage usually remains present across childhood. In families expereriencing social disadvantage where stress, available time and access to resources limit their ability to provide enriching home environments, again a single intervention is unlikely to fully mitigate this and families will need support across the pre‐school years.

Importantly, the magnitude of the effect of parental responsiveness decreased when the model was adjusted for effects of socio‐demographic variables and earlier language skills. This is a useful reminder that the effects of social disadvantage are not entirely mediated through parental responsiveness. Rather, there may be other, more distal or ‘upstream’ factors in the child's environment driving these inequalities in language outcomes. Therefore, it is vital that interventions do not focus solely on parental responsiveness, but also seek to reduce inequalities in more distal factors such as income, housing and parental education. It is also important to note that parent–child interactions are bi‐directional and less responsive parental behaviours could be a result of parent's recognition that their child is at a lower stage of language acquisition, that is, children with low language abilities provide their parents with less opportunities to provide responsive quality input (Weisleder & Fernald, 2013).

Preventative interventions aimed at promoting early language acquisition via enhanced parent–child interaction are widely used and there is evidence to support positive effects of this type of intervention for children's short‐term outcomes (Heidlage et al., 2020). As noted above, a key feature of these parent‐facilitated language interventions is promoting responsive parental behaviours (Jeong et al., 2021). Universally accessible platforms, such as maternal and child health services, could target interventions by considering both parent–child interaction and child language abilities to identify the need for support with responsive parenting. The difference demonstrated in the current study between the consistently high, inconsistent and consistently low responsive behaviours groups across the early years suggest that models of surveillance and support which monitor and support families at multiple time points over the early years are likely to be most effective. Such services should monitor the home learning environment and child language ability, consider possible changes in parents’ circumstances and, where needed, provide a series of interventions. Including appropriate measures, observations and/or parent reports of responsive parental behaviours, could enable a rapid response to support parents when they need advice and support. While the BPCIS items may reflect the interactional feature of caregiver quality input, it would also be worthwhile to capture measures of the linguistic and conceptual features of caregiver quality input, as identified by Rowe and Snow (2020). In addition, it would be beneficial to validate the BPCIS by examining agreement of the scale with direct observations of parent–child interaction. The BPCIS scores in the current sample were skewed towards higher use of responsive behaviours, suggesting many parents were self‐reporting use of responsive behaviours, which may be in part a reflection of the sample demographics. The current sample included on average mothers with higher education levels and families living in more socio‐economically advantaged areas, so it would be useful to include the BPCIS with families experiencing greater levels of disadvantage.

It is important to note that the current study does not demonstrate causality, that is, that use of responsive parental behaviours results in better child language outcomes. There is a need to further understand the bidirectional transactions between parent behaviours and child language acquisition, as well as the complex interplay between genetics and environment (Dale et al., 2015). The findings in the current study reflect this: the inclusion of early vocabulary skills to the final model increased the variance explained in the language outcome from 13% to 22%, but a large proportion of variance in language at age 7 years remained unexplained. Further research is needed into the contribution of other environmental factors, such as early childhood education (ECE) settings, that potentially act as a protective factor for those children who are not experiencing regular responsive parent–child interactions in the home environment. Exposure to quality ECE has been shown to moderate the association between the home language‐learning environment and children's language skills, suggesting a positive cumulative effect of home and ECE settings (Pinto et al., 2013). However, the quality of ECE programmes and family access to quality ECE programmes must be considered. Vulnerable and disadvantaged children, who are also at risk of suboptimal home environments, are shown to have poorer access to quality ECE programmes (Wong et al., 2014). Policy to improve and maintain the quality, reach and accessibility of ECE is therefore vital.

### Limitations

Although the original ELVS sample was recruited to be representative of the geographic and socio‐economic spectrum, attrition has led to the sample at age 7 years being more advantaged in terms of socio‐economic background and maternal education level. Therefore, the sample included in the current study is unlikely to represent the original ELVS sample, including more disadvantaged groups.

We were limited to self‐report of responsive parental behaviours used in ELVS. Direct observations are often viewed as the gold standard for measuring responsive behaviours. However, a major limitation of direct observations is that they are time and resource intensive, often making these methods unfeasible in large cohort studies and for use in universal surveillance or clinical practice. While there are limitations to the use of self‐report of parenting including response bias, strengths include the fact that they draw on parents’ own experience of interacting with their child and that they draw on aspects of interaction across a range of contexts. This potentially increases validity in that they represent more than a single snapshot in time but rather consider interactions that occur day to day and also include consideration across contexts, such as mealtimes, care routines, shared book reading and play (Herbers et al., 2017). Brief parent‐reported measures of responsive parental behaviours have demonstrated good agreement with observational measures, suggesting parent‐reported measures may be a reliable indicator of parental responsiveness and a feasible alternative to time‐intensive and costly observational methods (Bennetts et al., 2016). Despite the limitations of self‐report measures of responsive parental behaviours, the findings from this study suggest the possibility of the clinical application of low‐burden self‐report tools to facilitate the provision of a responsive pathway of support over the early years.

There are several factors that may account for differences between parent‐reported and direct measures of parent behaviours, including family SES, non‐English‐speaking background and parent symptoms of depression or distress (Bennetts, et al., 2016; Herbers et al., 2017). Families from a non‐English‐speaking background have been shown to have lower scores on direct observational measures compared with self‐reported measures (Bennetts, et al., 2016), while parents from low SES backgrounds and parents reporting symptoms of depression were scored more favourably by observers compared with self‐report of parenting behaviours (Herbers et al., 2017). To account for this, we adjusted for SES, maternal mental health and non‐English‐speaking background in our analyses.

## CONCLUSIONS

There is a wealth of evidence to suggest that early language development involves bi‐directionality, whereby parent behaviours influence child language development but parents’ behaviour is also modified by children's level of language. Consistency of responsive parental behaviours which are also sensitive to the child's developmental level over the pre‐school years are vitally important to both short‐ and longer term outcomes into the school years. We cannot take for granted that all parents will be able to modify their interactions intuitively to meet the rapidly changing developmental needs of their child nor that parents’ circumstances which might impact their ability to be contingent and responsive stay stable over the pre‐school years. There is also a clear need for surveillance of children and families in the early years, ensuring that intervention occurs when families need it most, that this support is responsive to changing needs and that nuanced advice and support strategies are provided to activate positive developmental cascades.

## CONFLICTS OF INTEREST

The authors report that they have no conflicts of interest.

## Data Availability

The participants of this study did not give written consent for their data to be shared publicly, so due to the sensitive nature of the research the supporting data are not available.
